# The Efficacy of Moxibustion for Breast Cancer Patients with Chemotherapy-Induced Myelosuppression during Adjuvant Chemotherapy: A Randomized Controlled Study

**DOI:** 10.1155/2021/1347342

**Published:** 2021-04-25

**Authors:** Yajie Ji, Siyu Li, Xinyue Zhang, Qiong Li, Qing Lu, Weili Chen, Yu Liu, Jiayu Sheng, Hongli Liang, Ke Jiang, Mengting Li, Shanyan Sha, Huangan Wu, Yan Huang, Xiaohong Xue

**Affiliations:** ^1^Department of Breast Surgery, Yueyang Hospital of Integrated Chinese and Western Medicine, Shanghai University of Traditional Chinese Medicine, Shanghai 200437, China; ^2^Shanghai Research Institute of Acupuncture and Meridian, Shanghai University of Traditional Chinese Medicine, Shanghai 200030, China; ^3^Key Laboratory of Acupuncture and Immunological Effects, Shanghai University of Traditional Chinese Medicine, Shanghai 200030, China

## Abstract

**Objective:**

The randomized controlled clinical trial aims to investigate the clinical efficacy of moxibustion for breast cancer patients with chemotherapy-induced myelosuppression (CIM) during adjuvant chemotherapy.

**Methods:**

Surgically resected breast cancer patients were randomly divided into the moxibustion group (MOX; *n* = 48) and control group (CON; *n* = 44). Routine adjuvant chemotherapy (every 21 days, 4–8 cycles) and supportive recombinant human granulocyte colony-stimulating factor were given to both groups, while MOX received an additional moxibustion treatment (once daily after each cycle of chemotherapy). Primary endpoints included the grade of myelosuppression in terms of white blood cell (WBC) and absolute neutrophil count (ANC) and the incidence of myelosuppression-related serious adverse events (SAEs). Other measures included treatment compliance, adverse events (AEs), and survival.

**Results:**

WBC counts were generally higher in MOX and were dramatically higher than those in CON at the 7^th^ course of chemotherapy (*P*=0.008), while grade 1 ANC reduction was dramatically lower than that in CON at the 7^th^course of chemotherapy (*P*=0.006). These effects were particularly significant in patients receiving anthracycline-taxane combination regimens. Moreover, MOX had fewer febrile neutropenia than CON (*P*=0.051). MOX demonstrated a lower incidence of grade 3–4 myelosuppression (*P* < 0.05). AEs including grade 2–3 severe nausea, various kinds of pains, and vertigo occurred less frequently in MOX (*P* < 0.05). No difference in survival was observed between the two groups (*P* > 0.05).

**Conclusion:**

Moxibustion is effective for treating CIM in breast cancer patients during adjuvant chemotherapy, especially for patients receiving high-dose, long-term, and combined chemotherapy regimens. Moxibustion can reduce the incidence of myelosuppression-related SAE and improve the compliance and safety of chemotherapy in breast cancer.

## 1. Introduction

Breast cancer is a chemotherapy-sensitive malignant disease [1]. Dose-dense anthracycline-taxane combination chemotherapy is usually recommended in early-stage patients [2] and is beneficial to overall survival in selected endocrine-sensitive patients [3]. However, this combination regimen causes high cytotoxicity to noncancerous normal tissue and leads to obvious chemotherapy-induced myelosuppression (CIM). Of which, febrile neutropenia (FN) is the major dose-limiting toxicity, which is associated with an increase in the risk of infection and can even be life-threatening [4, 5]. Hence, the prevention of CIM-related toxicities, as well as improving patients' quality of life (QoL), is the key to breast cancer management.

Myeloid growth factor (MGF) such as recombinant human granulocyte colony-stimulating factor (rhG-CSF) is currently used to treat and prevent CIM [6]. The use of MGF can improve disease-free survival (DFS) and overall survival (OS) in breast cancer [7]. The WBC-increasing effect is fast but rather short-lived. Moreover, 20–30% of the patients receiving MGF reported adverse events (AEs) and even serious adverse events (SAEs) including myelodysplastic syndrome [8] and acute myeloid leukemia [9]. These greatly hampered the patients' prognosis and QoL.

Traditional Chinese Medicine (TCM) that adjusts the body's Yin-Yang balance and emphasizes holistic concepts and pattern identification may be a potential alternative for MGF. Moxibustion is one of the TCM therapies that stimulate specific acupoints of the patient's body with dried moxa, intending to protect the health and prevent diseases [10]. Chen found that moxibustion can effectively prevent leukopenia and reduce gastrointestinal side effects after chemotherapy in breast cancer patients [11]. A recent study also revealed that moxibustion can improve the general conditions of patients with malignant tumors [12]. Given its convenience and low cost, moxibustion may be applied as a complementary treatment for breast cancer.

Currently, there is a lack of high-level clinical evidence (i.e., RCTs) in applying moxibustion to relieve CIM in breast cancer [13]. The consensus on choices of acupoints, methods, and course of moxibustion treatment is urgently warranted. Therefore, we aimed to systematically evaluate the effect of moxibustion for breast cancer patients receiving adjuvant chemotherapy in a randomized controlled setting.

## 2. Materials and Methods

### 2.1. Study Design

This is a randomized controlled trial (RCT) conducted at Shanghai Yueyang Integrated Traditional Chinese Medicine and Western Medicine Hospital. Postoperative breast cancer patients who were expected to receive adjuvant chemotherapy were recruited during 2015 and 2016. Inclusion criteria included the following: (1) diagnosed with breast cancer according to both Chinese and Western standards, (2) female, (3) aged between 18 and 80 years old, (4) pathologically confirmed primary breast cancer, (5) patients expected to receive adjuvant chemotherapy according to the “CACA Guidelines for Breast Cancer Diagnosis and Treatment (2015 edition)”, (6) agreed to complete a full course of chemotherapy, (7) good compliance, and (8) written informed consent obtained. Exclusion criteria included the following: (1) history of receiving systemic chemotherapy or radiotherapy, (2) abnormal liver and kidney functions, serum alanine aminotransferase (ALT)/aspartate aminotransferase (AST)  ≥2.5 times higher than the normal upper limit, and serum creatine (sCr) ≥150 *μ*mol/L, (3) major organ dysfunction such as severe heart disease, (4) coexistence of other primary malignancies, (5) pregnant or lactating women (according to blood *β*-HCG test), (6) language or geographic issues that affect the follow-up of patients, and (7) other conditions that may cause risks to the patients or confuse the results as determined by the investigators. Eligible patients were randomly assigned into the moxibustion group (MOX) which received an additional moxibustion treatment and the control group (CON) which received the routine adjuvant chemotherapy without moxibustion. Primary endpoints included the grade of myelosuppression in terms of white blood cell (WBC) and absolute neutrophil count (ANC) and the incidence of myelosuppression-related SAE. Other measures included treatment compliance, AE, DFS, and OS.

This study was approved by the Ethics Committee of the Shanghai Yueyang Integrated Traditional Chinese Medicine and Western Medicine Hospital (Approval number: 2015–050) and was conducted in accordance with the Declaration of Helsinki. Written informed consent was obtained from each participant before study enrollment.

### 2.2. Diagnostic Standards

For the Western diagnostic standards, clinical diagnosis was based on the Tumor of the Breast (2^nd^ Edition) edited by Shao Zhimin; the pathological diagnosis was based on the WHO Classification of Tumors of the Breast (4^th^ Edition); the staging was based on the American Joint Committee on Cancer Staging Form (8^th^ Edition). For the Chinese diagnostic standards, the diagnosis was based on the Practical Surgery of Traditional Chinese Medicine (2^nd^ Edition).

### 2.3. Sample Size Estimation

According to our preliminary study, the occurrence rates of grade 2 or above myelosuppression in MOX and CON were 39% and 81%, respectively. Patients were randomly assigned to the two groups at a 1 : 1 ratio. The Z-pooled normal approximation was used to estimate the required sample size. Assuming that the two-sided significance level (*α*) is 0.05 and the power (1-*β*) is 0.2, the calculated sample size was 44 cases in MOX and 44 cases in CON.

## 3. Method for Randomization

A stratified randomization method was applied in this study using the online clinical trial random grouping management system which automatically assigned participants into groups after data entry. Patients were stratified according to the received chemotherapy regimens (in particular, anthracycline-only, taxane-only, and anthracycline-taxane combination chemotherapy). The administrative staff was responsible for generating the random allocation sequence and providing the treatment allocation to the physicians in sealed envelopes. The physicians were then responsible for assigning participants to corresponding interventions.

### 3.1. Moxibustion Treatment

Shenque (CV8), bilateral Zusanli (ST36), and bilateral Sanyinjiao (SP6) were selected as the acupoints for moxibustion based on the Science of Meridian and Acupuncture [14]. Shenque is located in the umbilicus. Zusanli is located in the outer side of the legs, 3 inches below Dubi (ST35), and between Dubi (ST35) and Jiexi (ST41). Sanyinjiao is located in the inner side of the legs, 3 inches above the tip of the medial malleolus, and at the posterior margin of medial tibia.

Mild moxibustion was applied to fully expose the above-selected acupoints. Hanyi® Pure Moxa Sticks for Mild Moxibustion was purchased from Nanyang Ltc. The moxa sticks were cut into 1.5 cm long to ensure constant size and density. When performing moxibustion, the lighted moxa stick was held accurately at the acupoint (about 2–3 cm away from the skin) so that the subject can feel warm but not burning. The skin areas applied with moxibustion should appear red. The subject first took a lying position for moxibustion at the Shenque and then took a sitting position for moxibustion at the bilateral Zusanli, followed by the bilateral Sanyinjiao. Moxibustion at each acupoint lasted for 15 minutes, followed by a gentle massage of the acupoints.

Moxibustion was performed once daily in MOX, started on the second day after each cycle of chemotherapy, and continued until the day before the next chemotherapy cycle. No moxibustion was planned on the days receiving chemotherapy. Moxibustion was applied until 3 weeks after the last cycle of chemotherapy. The exact treatment course depended on the chemotherapy cycles which were 4–8 cycles in this study.

### 3.2. Chemotherapy Regimen

Both MOX and CON received adjuvant chemotherapy treatment for breast cancer (every 21 days). Anthracycline-only, taxane-only, or anthracycline-taxane combination regimen was administered as decided by the investigators according to the CACA Guidelines for Breast Cancer Diagnosis and Treatment (2015 edition) [15]. rhG-CSF was applied to patients according to NCCN Clinical Practice Guidelines in Oncology: Myeloid Growth Factors (Version 1.2015) [16]. In brief, patients were closely followed up when grade 1 myelosuppression occurred. For patients with grade 2 or above myelosuppression, rhG-CSF was applied until their WBC counts and ANC returned to normal.

General supportive care for preventing and curing other chemotherapy-related AEs and certain medications that must be taken for patients with comorbidities were allowed as concurrent treatments in this study. However, Chinese herbal medicinal extracts and other TCM treatments for treating myelosuppression were prohibited.

### 3.3. Laboratory Tests and Technical Examination

Routine blood tests were performed at the first (day 1–7), second (day 8–14), and third (day 15–20) weeks after each cycle of chemotherapy (every 21 days). Parameters including WBC counts, ANC, hemoglobin (HGB), and platelet (PLT) were recorded. The reductions in WBC counts and ANC were compared between MOX and CON. Besides, liver functions including alanine transaminase (ALT), aspartate transaminase (AST), and *γ*-glutamyltranspeptidase (GGT); kidney functions including serum creatinine (sCr); blood lipids including triglyceride (TG) and total cholesterol (TC); heart responses including creatine kinase (CK), and left ventricular ejection fraction (LVEF) were also recorded.

### 3.4. Adverse Effects

The incidences of common adverse effects such as general systemic conditions, gastrointestinal reactions, and pain were recorded. Particularly, the incidence rates of CIM-related SAEs including the proportion of patients with FN, grade 3-4 WBC counts and/or ANC reduction, and grade 3-4 abnormal laboratory indicators of the two groups were compared. Moxibustion-related AEs including skin reactions, burns, and heat symptoms were also recorded. The grading was determined according to the CTCAE 4.0.2.

### 3.5. Chemotherapy Compliance

Poor compliance was defined as a shortened course of chemotherapy and/or a reduction in chemotherapy dose. The proportion of patients who complete the standard course of treatment with standard dosing was compared between the two groups.

### 3.6. Survival Analysis

Follow-up was performed by the investigator through face-to-face interviews, telephone calls, and a WeChat management platform (ID: yanyueesf). The WeChat management platform was provided by Shanghai Ruochu Information and Technology Co. Ltd. The company has signed a nondisclosure agreement to protect the participants' confidentiality. DFS was defined as the time from the day of randomization to the day of disease progression or death, while OS was defined as the time from the day of randomization to the day of death. Kaplan–Meier method was used to generate the survival curves and calculate the survival rates. A Rank-sum test was used to compare the survival rates between the two groups.

### 3.7. Statistical Analysis

Two datasets were generated for subsequent analysis: a full analysis set (FAS) including all selected intent-to-treat (ITT) populations and a per-protocol set (PPS) including the per-protocol (PP) populations among the selected ones who completed the trial. Baseline and survival analyses were performed using the FAS, while the other data analyses were performed using the PPS.

Data analysis was performed using SPSS 22.0 software. All analysis was two-tailed and a *P* value of less than 0.05 was regarded statistically significant. Continuous variables that follow normal distribution were compared by using independent *t*-tests or ANOVA; otherwise, the Kruskal-Wallis H test and Nemenyi test were used. For categorical variables, the Chi-square test or Fisher's exact test was used for comparison.

## 4. Results

### 4.1. Demographic Information

From July 1, 2015, to July 31, 2016, a total of 132 postoperative breast cancer patients were recruited. 98 of them (49 in MOX and 49 in CON) were included as the FAS population and 92 of them (48 in MOX and 44 in CON) were included as the PPS population (Supplementary Figure 1). The ITT population showed no significant statistical difference (Supplementary Table 1) in basic information, and the PPS population was also comparable at baseline (Supplementary Table 2).

### 4.2. Effects of Moxibustion on Myelosuppression

Generally, during the whole course of chemotherapy, no significant difference (*P* > 0.05) was found in the incidence of grade 0–4 WBC count reduction, ANC reduction, HGB reduction, and PLT reduction between the two groups (Supplementary Table 3).

Next, we compared the WBC counts of the two groups at specific time points (Table 1). There was no significant difference in WBC counts in the first, second, and third weeks after cycle 1–5 chemotherapy treatment between MOX and CON. But as the course of chemotherapy progressed, the difference in WBC counts began to occur. The WBC count of MOX in the first week after cycle 6 chemotherapy was slightly higher than that of the CON (*P* = 0.084). In the 7^th^ cycle, the WBC count in the first week after chemotherapy was significantly higher in MOX (5.38 ± 1.41 vs 3.78 ± 1.46 × 10^9^/L, *P* < 0.01). However, the difference returned nonsignificant in the 8^th^cycle of treatment. We also found no difference in the incidence of grade 0–4 WBC count reduction between the two groups (Supplementary Table 4).

Throughout the courses of chemotherapy, there was no significant difference in ANC between MOX and CON (Supplementary Table 5). However, a trend of a higher percentage of grade 0 ANC patients was observed in cycle 5 (79.4% vs 60.6%, *P*=0.057) and cycle 7 (75.0% vs 45.5%, *P*=0.051) in the MOX group. In addition, grade 1 ANC reduction in MOX was significantly lower than that in CON (0% vs 31.8%, *P* < 0.01; Table 2) in cycle 7.

### 4.3. Stratified Analysis according to the Applied Chemotherapy Regimens

**Figure 1:**
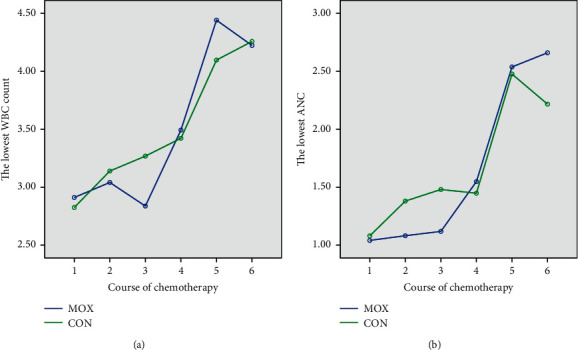
Comparisons of the lowest WBC counts and ANC between the two groups. Line charts showing (a) the lowest WBC counts and (b) the lowest ANC of the MOX and CON groups during the course of chemotherapy.

The majority of the patients (65/92, 70.7%) received an anthracycline-taxane combination regimen in this study, including 32 patients in the MOX group and 33 patients in the CON group. According to Figures 1(a) and 1(b), the lowest WBC counts and ANC in the MOX group were higher compared to the CON group since the 4^th^ cycle of chemotherapy.

**Figure 2:**
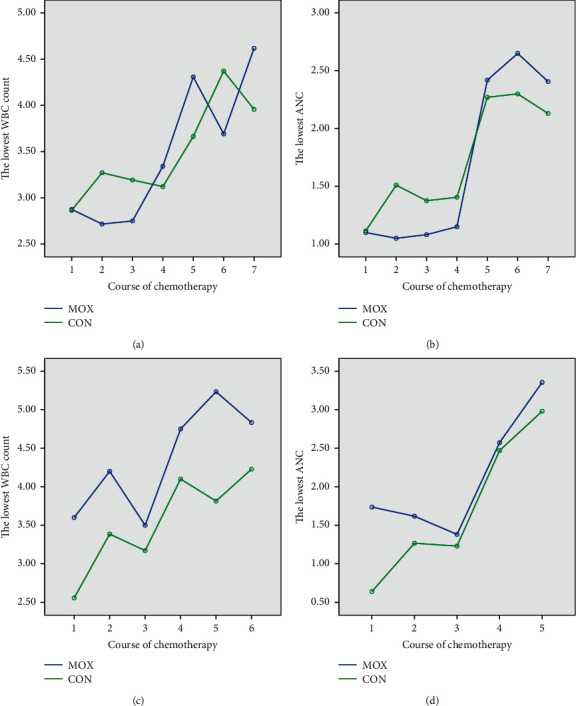
Comparisons of the lowest WBC counts and ANC in the subgroup of patients who received 8-course chemotherapy. Line charts showing (a) the lowest WBC counts and (b) the lowest ANC of the MOX and CON groups during the course of chemotherapy. Comparisons of the lowest WBC counts and ANC in the subgroup of patients who received 6-course chemotherapy. Line charts showing (c) the lowest WBC counts and (d) the lowest ANC of the MOX and CON groups during the course of chemotherapy.

Further analysis stratifying the patients who received an anthracycline-taxane combination regimen according to the total course of treatment was performed. For the 44 patients who received 8-course of chemotherapy (21 in MOX and 23 in CON), the lowest WBC counts in the MOX group were higher than those of the CON group in cycles 4, 5, and 7 (Figure 2(a)). The MOX group also showed a higher value of the lowest ANC compared to the CON group since cycle 5 of chemotherapy (Figure 2(b)). For the 21 patients who received 6-course of chemotherapy (11 in MOX and 10 in CON), the lowest WBC counts and the lowest ANC in the MOX group were higher than those of the CON group at any time point (Figures 2(c) and 2(d)).

Other than the anthracycline-taxane combination regimen, 5 patients (5.4%; 2 in MOX and 3 in CON) received an anthracycline-only regimen and 22 patients (23.9%; 14 in MOX and 8 in CON) received a taxane-only regimen in this study, respectively. No statistically significant differences in the lowest WBC count and the lowest ANC were found between the anthracycline-only group and the taxane-only group.

### 4.4. Effects of Moxibustion on AEs

There were no significant differences observed in liver and kidney functions (ALT elevation, AST elevation, GGT elevation, and sCr elevation), blood lipids (TG elevation and TC elevation), and cardiac response (CK elevation and LVEF reduction) between the two groups (Supplementary Table 6).

No significant differences in fatigue, malaise, and weight loss were observed between the two groups. Trends of fewer cases of grade 2 fatigue (12.5% vs 20.5%) and grade 2 malaise (8.3% vs 15.9%) were observed in the MOX group compared to the CON group (Supplementary Table 7).

For gastrointestinal reactions, grade 2–3 severe nausea in the MOX group was significantly less frequent compared to the CON group (27.1% vs 47.7%, *P* < 0.05). A trend of fewer cases of vomiting, diarrhea, and constipation was also observed in the MOX group (Supplementary Table 8).

The incidence of pain was slightly lower in the MOX group (89.6% vs 97.7%). Subdividing this into different sites of pain, we observed that bone, joint, muscle, and incision pain were less frequent in the MOX group (*P* < 0.05; Supplementary Table 9).

The cases of hot flashes were higher in the MOX group (25.0% vs 11.4%, *P*=0.092), but the proportion of vertigo was significantly less than that in the CON group (4.2% vs 27.3%, *P* < 0.01; Supplementary Table 10).

No moxibustion-related toxic side effects including skin reactions, burns, and heat syndrome were observed in the MOX group.

### 4.5. Chemotherapy Compliance

For myelosuppression-related SAE, the number of FN cases in the MOX group was fewer than that in the CON group (1 vs 6, *P* = 0.051); the number of grade 3–4 severe myelosuppression cases (WBC count and/or ANC reduction) in the MOX group was significantly fewer than that in the CON group (30 vs 36, *P* < 0.05); no significant difference in the incident of grade 3 ALT and/or AST elevation was found between the two groups.

The above-mentioned SAEs may cause the early termination of chemotherapy; however, the number of cases that terminated chemotherapy earlier than expected showed no difference between the two groups (Supplementary Table 11). In contrast, the MOX group had a slightly lower chance of dose reduction compared to the CON group (2.1% vs 11.4%, *P*=0.100).

### 4.6. Survival Analysis

All the 98 patients in the FAS population were successfully followed up with a median follow-up duration of 45.1 months (38.0–51.6 months). Distant recurrence was observed in 6 cases (6.1%) with a median time to distant recurrence of 22.9 months (13.0–25.2 months). Death was observed in 2 cases (2.0%), with a survival time of 31.1 and 33.2 months, respectively.

**Figure 3:**
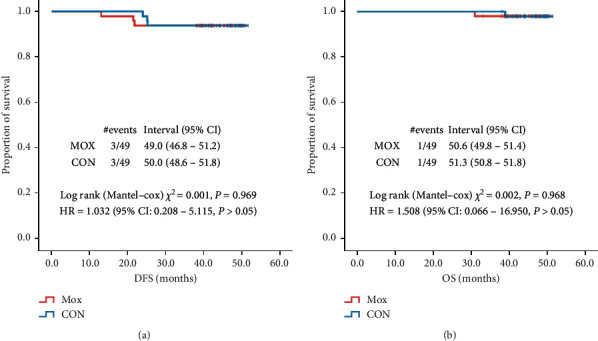
Comparisons of the DFS and OS between the two groups. Kaplan-Meier curves showing (a) the DFS and (b) the OS of the MOX and CON groups.

According to the Kaplan-Meier analysis shown in Figure 3(a), the median DFS in MOX and CON was 49.0 months (95% CI: 46.8–51.2 months) and 50.0 months (95% CI: 48.6–51.8 months), respectively. The median OS in MOX and CON was 50.6 months (95% CI: 49.8–51.4 months) and 51.3 months (95% CI: 50.8–51.8 months), respectively (Figure 3(b)). The DFS and OS were comparable between the two groups (*P* > 0.05).

The analysis of the sites of distant recurrence revealed 5 cases of lung metastasis and 1 case of liver metastasis, with 1 case of death in each group. However, there were 2 cases in MOX that standard treatments were not given due to economic factors (Supplementary Table 12).

## 5. Discussion

Postoperative CIM in breast cancer often causes fatigue, dizziness, and unwillingness to speak, palpitations, insomnia and dreaminess, spontaneous sweating and tachypnea, pale and enlarged tongue, weak pulses, etc. Jiang has proven that the effect of moxibustion comes from the warm and hot stimulus produced by the burning of moxa [17] so that the neuroendocrine-immune network can be gradually regulated.

Previous clinical studies showed that moxibustion can increase the WBC count for 55.9–84.0% and improve the patients' QoL [18–22]. However, the physicians have not yet come to a consensus on the acupoints, methods, courses, and volume of moxibustion. The evidence supporting moxibustion on CIM is of low quality. Further research is needed to provide a well-evaluated moxibustion protocol for supportive treatment in breast cancer patients.

The primary outcome measures of this study were WBC count and ANC. Different chemotherapy regimens and courses of treatment may also influence the blood test results [23]. Therefore, we compared the WBC counts and ANC at different time points during chemotherapy and carried out stratified analysis according to chemotherapy regimens. Consistent with Chen's and Jiang's conclusion [11, 24], our results revealed that the WBC counts and ANC reduction grade gradually showed a slight difference between the two groups as the course of chemotherapy progressed, indicating that moxibustion can stabilize the WBC count and ANC in the latter course of the chemotherapy. Although moxibustion did not show significant advantages for patients receiving anthracycline-only and taxane-only regimens, beneficial effects were observed in patients receiving combination regimens, especially for those receiving high-dose and multiple courses of chemotherapy. Interestingly, it is found that WBC count and ANC reduction grade are more sensitive in evaluating moxibustion on CIM.

In this study, there were no significant differences in the occurrence of gastrointestinal-related AEs between the two groups. However, the incidence of grade 2–3 severe nausea in MOX was significantly less than that in CON, indicating that moxibustion has an effect on preventing nausea and vomiting. Consistent with our findings, Zhao had also reported that moxibustion at Shenque and Zusanli acupoints can prevent gastrointestinal reactions caused by chemotherapy [25].

With the help of the WeChat management platform, the follow-up rate of the FAS population was 100% (98/98). The median follow-up duration is 45.1 (38.0–51.6) months. The occurrence rates of events were lower than those previously reported [26]. Six cases of patients who had survival events showed a median time to distant recurrence of 22.9 months, which seems logical regarding that the peak period of distant recurrence is 2–3 years after surgical treatment in breast cancer [27]. Survival data showed that moxibustion is not detrimental to the patients' DFS and OS.

## 6. Conclusions

This RCT study suggested that moxibustion could prevent CIM in breast cancer patients, especially in the 7^th^ course of chemotherapy. For patients receiving high-dose and multiple courses of anthracycline-taxane combined chemotherapy, the efficacy of moxibustion was particularly obvious. Additionally, moxibustion may lower the incidence of SAE (in myelosuppression), reduce AE (such as nausea, vertigo, bone, joint, and muscle pain, and incision pain), and increase compliance and safety of chemotherapy. In this RCT, our results provided systematic evidence for the use of a simple, convenient, and inexpensive (relatively, compared to rhG-CSF) moxibustion protocol to relieve CIM in breast cancer patients receiving adjuvant chemotherapy. A study of the combination therapy of moxibustion and Traditional Chinese Medicine is ongoing [28].

## Figures and Tables

**Table 1:** Comparison of the WBC counts at specific time points between the two groups.

Chemotherapy course	Group	n	WBC count		
			1^st^ week after chemotherapy (1–7 d)	2^nd^ week after chemotherapy (8–14 d)	3^rd^ week after chemotherapy (15–20d)
1^st^ course	MOX	48	5.03 ± 1.93 (36)	3.25 ± 1.76 (45)	6.67 ± 2.12 (47)
	CON	44	4.61 ± 1.68 (36)	3.11 ± 1.31 (39)	6.33 ± 2.35 (44)

2^nd^ course	MOX	48	5.08 ± 1.70 (38)	3.41 ± 1.42 (44)	5.86 ± 1.67 (46)
	CON	44	5.33 ± 2.07 (33)	3.21 ± 1.12 (38)	5.47 ± 1.85 (44)

3^rd^ course	MOX	48	4.92 ± 1.68 (35)	3.50 ± 1.25 (44)	5.50 ± 1.85 (45)
	CON	44	4.74 ± 1.98 (34)	3.22 ± 1.44 (40)	5.19 ± 1.15 (44)

4^th^ course	MOX	48	4.86 ± 1.94 (23)	3.51 ± 1.61 (33)	5.71 ± 1.63 (38)
	CON	44	5.04 ± 1.82 (25)	3.66 ± 2.17 (33)	5.22 ± 2.74 (36)

5^th^ course	MOX	33	5.22 ± 2.74 (19)	4.72 ± 1.63 (29)	5.79 ± 1.66 (33)
	CON	34	5.05 ± 2.11 (23)	5.20 ± 1.84 (25)	6.15 ± 1.79 (34)

6^th^ course	MOX	33	6.40 ± 2.00 (20) ^#^	4.33 ± 2.09 (21)	5.40 ± 1.75 (24)
	CON	34	4.33 ± 2.09 (14) ^#^	4.71 ± 1.27 (22)	5.42 ± 1.42 (14)

7^th^ course	MOX	20	5.38 ± 1.41 (12) ^*∗∗*^	4.87 ± 1.82 (18)	5.08 ± 1.42 (20)
	CON	23	3.78 ± 1.46 (15) ^*∗∗*^	4.67 ± 1.79 (20)	5.21 ± 1.01 (21)

8^th^ course	MOX	20	5.19 ± 2.43 (9)	5.35 ± 2.44 (8)	5.03 ± 1.66 (7)
	CON	23	3.95 ± 1.27 (6)	4.19 ± 1.04 (8)	5.86 ± 1.07 (7)

WBC counts are expressed as mean ± standard deviation x10^9^/L (number of cases). ^#^*P*=0.084,^ *∗∗*^*P* < 0.01.

**Table 2:** Comparison of the ANC count reduction grading between the two groups.

Chemotherapy course	Group	n	ANC reduction grading				
			Grade 0	Grade 1	Grade 2	Grade 3	Grade 4
1^st^ course	MOX	48	15 (31.3)	3 (6.3)	8 (16.7)	15 (31.3)	7 (14.6)
	CON	44	10 (22.7)	5 (11.4)	6 (13.6)	13 (29.5)	10 (22.7)

2^nd^ course	MOX	48	15 (31.3)	4 (8.3)	16 (33.3)	11 (22.9)	2 (4.2)
	CON	44	14 (31.8)	7 (15.9)	7 (15.9)	15 (34.1)	1 (2.3)

3^rd^ course	MOX	48	16 (33.3)	3 (6.3)	14 (29.2)	12 (25.0)	3 (6.3)
	CON	43	14 (32.6)	2 (4.7)	13 (30.2)	11 (25.6)	3 (7.0)

4^th^ course	MOX	42	22 (52.4)	2 (4.8)	8 (19.0)	6 (14.3)	4 (9.5)
	CON	37	13 (35.1)	5 (13.5)	7 (18.9)	10 (27.0)	2 (5.4)

5^th^ course	MOX	34	27 (79.4) ^#^	3 (8.8)	1 (2.9)	2 (5.9)	1 (2.9)
	CON	33	20 (60.6) ^#^	1 (3.0)	4 (12.1)	5 (15.2)	3 (9.1)

6^th^ course	MOX	27	18 (66.7)	5 (18.5)	2 (7.4)	1 (3.7)	1 (3.7)
	CON	25	16 (64.0)	1 (4.0)	3 (12.0)	3 (12.0)	2 (8.0)

7^th^ course	MOX	20	15 (75.0) ^*∗*^	0 (0.0) ^*∗∗*^	2 (10.0)	1 (5.0)	2 (10.0)
	CON	22	10 (45.5) ^*∗*^	7 (31.8) ^*∗∗*^	2 (9.1)	2 (9.1)	1 (4.5)

8^th^ course	MOX	12	10 (83.3)	1 (8.3)	1 (8.3)	0 (0.0)	0 (0.0)
	CON	13	7 (53.8)	3 (23.1)	2 (15.4)	0 (0.0)	1 (7.7)

Variables are expressed as the number of cases (percentage). ^#^*P*=0.057,^*∗*^*P*=0.051,^*∗∗*^*P* < 0.01.

## Data Availability

All the study data are available upon reasonable request.

## Abbreviation

E: Epirubicin
C: Cyclophosphamide
T: Taxol
F: Fluorouracil
H: Herceptin
AI: Aromatase inhibitor
OFS: Ovarian function suppression
TAM: Tamoxifen.

## Supplementary Materials

Supplementary Materials Supplementary Figure 1: flow chart of patient enrollment. Supplementary Table 1: baseline characteristics of the FAS population. Variables are expressed as either mean ± standard deviation or number of cases (percentage). Supplementary Table 2: baseline characteristics of the PPS population. Variables are expressed as either mean ± standard deviation or number of cases (percentage). Supplementary Table 3: comparison of myelosuppression occurred during chemotherapy between the two groups. Supplementary Table 4: comparison of the WBC count reduction grading between the two groups. Variables are expressed as number of cases (percentage). Supplementary Table 5: comparison of the ANCs at specific time points between the two groups. ANCs are expressed as mean ± standard deviation x10^9^/L (number of cases). Supplementary Table 6: comparison of adverse effects between the two groups. Variables are expressed as number of cases (percentage). Supplementary Table 7: comparison of systemic general adverse effects between the two groups. Variables are expressed as number of cases (percentage). Supplementary Table 8: comparison of gastrointestinal adverse effects between the two groups. Variables are expressed as number of cases (percentage). ^*∗*^*P* < 0.05. Supplementary Table 9: comparison of the pain adverse effects between the two groups. Variables are expressed as number of cases (percentage). Musculoskeletal and connective tissue disorders include bone pain, arthralgia, and muscle pain. ^*∗*^*P* < 0.05. Supplementary Table 10: comparison of the other adverse effects between the two groups. Variables are expressed as number of cases (percentage). ^#^*P* = 0.092,^*∗∗*^*P* < 0.01. 1: infection includes oral mucositis, rhinitis, pneumonia, cholecystitis, gastroenteritis, pelvic inflammatory disease, incision infection, and urinary tract infection. 2: paresthesia includes dysesthesia, dysgeusia, scalp numbness, and general numbness. 3: edema includes facial edema, pedal edema, and periocular edema. 4: hemorrhage includes bleeding gums, nosebleed, and subconjunctival hemorrhage. 5: surgical and medical procedures include 1 case of venous thrombosis and 2 cases of breast surgery. Supplementary Table 11: comparison of the chemotherapy compliance between the two groups. Variables are expressed as number of cases (percentage). Supplementary Table 12: clinical characteristics of the 6 cases with survival events observed. ∗Death interval is defined as the time between distant recurrence and death.
