# Supplementary material for: The Efficacy of Moxibustion for Breast Cancer Patients with Chemotherapy-Induced Myelosuppression during Adjuvant Chemotherapy: A Randomized Controlled Study
Source: Evid Based Complement Alternat Med. 2021 Apr 25;2021:1347342. doi: 10.1155/2021/1347342 (PMC8093063; doi:10.1155/2021/1347342)
Supplement: Supplementary Materials — Supplementary Figure 1: flow chart of patient enrollment. Supplementary Table 1: baseline characteristics of the FAS population. Variables are expressed as either mean ± standard deviation or number of cases (percentage). Supplementary Table 2: baseline characteristics of the PPS population. Variables are expressed as either mean ± standard deviation or number of cases (percentage). Supplementary Table 3: comparison of myelosuppression occurred during chemotherapy between the two groups. Supplementary Table 4: comparison of the WBC count reduction grading between the two groups. Variables are expressed as number of cases (percentage). Supplementary Table 5: comparison of the ANCs at specific time points between the two groups. ANCs are expressed as mean ± standard deviation x109/L (number of cases). Supplementary Table 6: comparison of adverse effects between the two groups. Variables are expressed as number of cases (percentage). Supplementary Table 7: comparison of systemic general adverse effects between the two groups. Variables are expressed as number of cases (percentage). Supplementary Table 8: comparison of gastrointestinal adverse effects between the two groups. Variables are expressed as number of cases (percentage). ∗P < 0.05. Supplementary Table 9: comparison of the pain adverse effects between the two groups. Variables are expressed as number of cases (percentage). Musculoskeletal and connective tissue disorders include bone pain, arthralgia, and muscle pain. ∗P < 0.05. Supplementary Table 10: comparison of the other adverse effects between the two groups. Variables are expressed as number of cases (percentage). #P = 0.092,∗∗P < 0.01. 1: infection includes oral mucositis, rhinitis, pneumonia, cholecystitis, gastroenteritis, pelvic inflammatory disease, incision infection, and urinary tract infection. 2: paresthesia includes dysesthesia, dysgeusia, scalp numbness, and general numbness. 3: edema includes facial edema, pedal edema, and periocular edema. 4: [file 1347342.f1.pdf]

SUPPLEMENTARY MATERIALS

Supplementary Fig. 1: Flow chart of patient enrollment

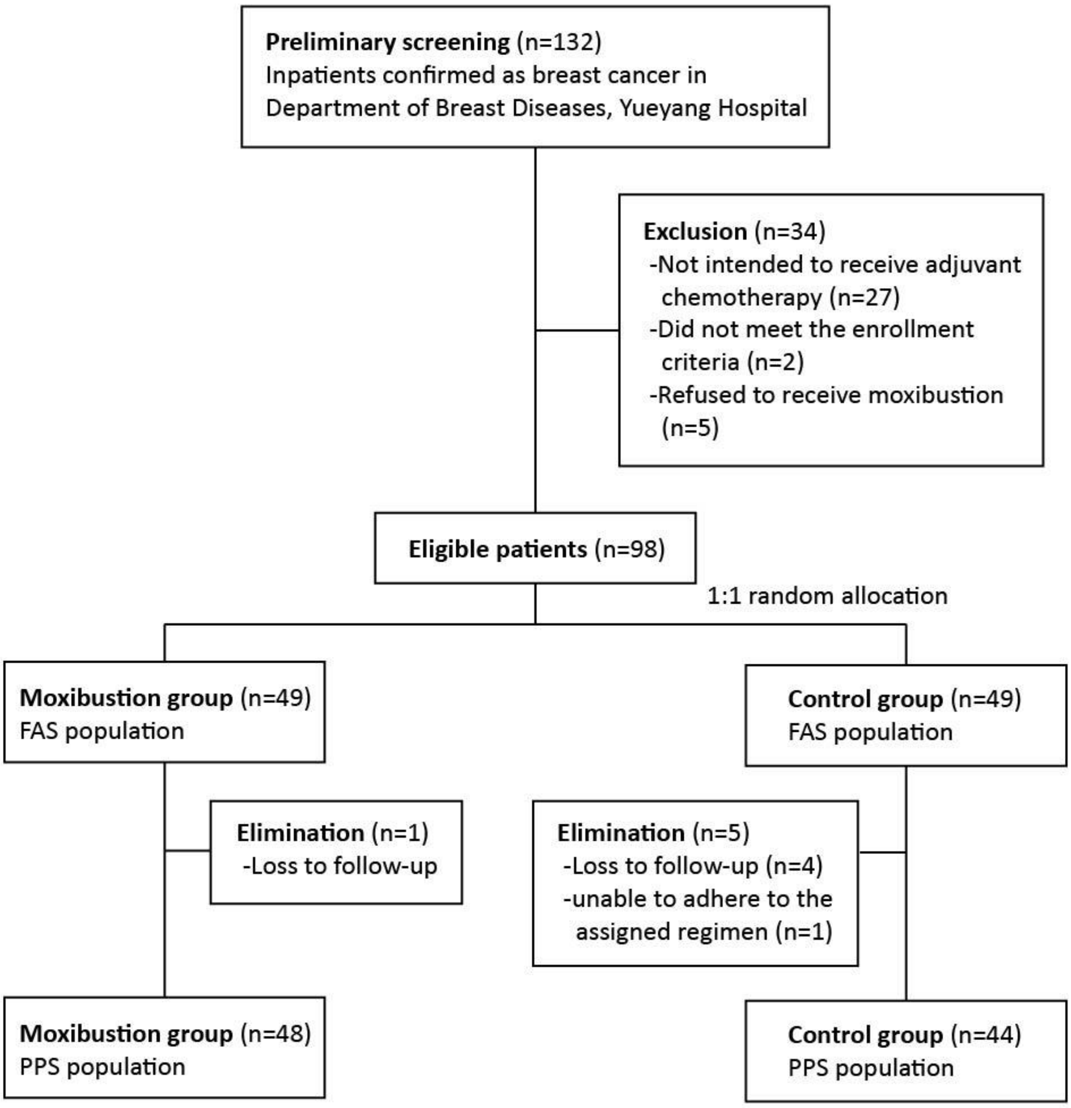

Supplementary Table 1: Baseline characteristics of the FAS population

Basic information	MOX (n=49)	CON (n=49)	t/χ^2	*P*
Age				
Mean±standard deviation	54.5±10.1	55.1±9.7	-0.276	0.783
Range	26-75	33-70		

Age group				
<40	4 (8.2)	3 (6.1)	0.154	0.695
40-59	27 (55.1)	26 (53.1)	0.041	0.839
≥60	18 (36.7)	20 (40.8)	0.172	0.678
Menstrual status				
Premenopause	20 (40.8)	18 (36.7)	0.172	0.678
Postmenopause	29 (59.2)	31 (63.3)	0.172	0.678
Marital status				
Unmarried	1 (2.0)	1 (2.0)	-	-
Married	48 (98.0)	48 (98.0)	-	-
Chemotherapy regimen				
Anthracycline-only	3 (6.1)	3 (6.1)	-	-
Taxane-only	14 (28.6)	10 (20.4)	0.883	0.347
Anthracycline- taxane	32 (65.3)	36 (73.5)	0.186	0.667
Chemotherapy course				
4-course	15 (30.6)	14 (28.6)	0.049	0.825
6-course	13 (26.5)	11 (22.4)	0.221	0.638
8-course	21 (42.9)	24 (49.0)	0.370	0.543
Surgical approach				
Breast conserving surgery	9 (18.4)	9 (18.4)	-	-
Mastectomy	40 (81.6)	40 (81.6)	-	-
Staging procedure				
Sentinel-node biopsy	15 (30.6)	18 (36.7)	0.411	0.521
Axillary dissection	34 (69.4)	31 (63.3)	0.411	0.521
Pathological staging				
I	20 (40.8)	18 (36.7)	0.172	0.678
II	24 (49.0)	24 (49.0)	-	-
III	5 (10.2)	7 (14.3)	0.380	0.538
Molecular subtype				

Luminal A	10 (20.4)	10 (20.4)	-	-
Luminal B (HER2 negative)	15 (30.6)	9 (18.4)	1.986	0.159
Luminal B (HER2 positive)	6 (12.2)	8 (16.3)	0.333	0.564
HER2 positive	12 (24.5)	11 (22.4)	0.057	0.812
Basal-like	6 (12.2)	11 (22.4)	1.779	0.182
Blood test				
WBC (x10^9/L)	6.4±1.5	6.2±1.3	0.927	0.356
ANC (x10^9/L)	4.3±1.4	3.9±1.0	1.743	0.085
HBG (g/L)	123.5±21.0	123.5±12.0	-0.002	0.998
PLT (x10^9/L)	213.1±52.8	193.7±51.6	1.841	0.069
Liver and kidney functions				
ALT (U/L)	21.6±25.5	18.0±8.9	0.938	0.351
AST (U/L)	19.5±4.6	19.5±6.3	0.018	0.985
GGT (U/L)	25.8±17.0	25.9±20.3	-0.027	0.979
sCr (μmol/L)	52.1±13.5	51.4±11.3	0.308	0.759
Blood lipids				
TG (mmol/L)	1.2±0.7	1.5±1.0	-1.939	0.056
TC (mmol/L)	4.9±1.1	4.8±1.2	0.648	0.518
Cardiac function				
CK (U/L)	76.7±35.2	74.9±30.5	0.270	0.788
LVEF (%)	70.8±6.2	69.5±5.3	0.796	0.430

Variables are expressed as either mean±standard deviation or number of case (percentage).

Supplementary Table 2: Baseline characteristics of the PPS population

Basic information	MOX (n=48)	CON (n=44)	t/χ^2	*P*
Age				
Mean±standard deviation	54.2±10.0	54.1±9.7	0.025	0.980

Range	26-75	33-70		
Age group				
<40	4 (8.3)	3 (6.8)	0.075	0.784
40-59	27 (56.3)	25 (56.8)	0.003	0.956
≥60	17 (35.4)	16 (36.4)	0.009	0.925
Menstrual status				
Premenopause	20 (41.7)	18 (40.9)	0.005	0.941
Postmenopause	28 (58.3)	26 (59.1)	0.005	0.941
Marital status				
Unmarried	1 (2.1)	1 (2.3)	0.004	0.95
Married	47 (97.9)	43 (97.7)	0.004	0.95
Chemotherapy regimen				
Anthracycline-only	2 (4.2)	3 (6.8)	0.314	0.575
Taxane-only	14 (29.26)	8 (18.2)	1.522	0.217
Anthracycline- taxane	32 (66.7)	33 (75.0)	0.769	0.381
Chemotherapy course				
4-course	15 (31.3)	10 (22.7)	0.843	0.359
6-course	13 (27.1)	11 (25.0)	0.052	0.820
8-course	20 (41.7)	23 (52.3)	1.037	0.308
Surgical approach				
Breast conserving rate	9 (18.8)	9 (20.5)	0.042	0.837
Mastectomy	39 (81.2)	35 (79.5)	0.042	0.837
Staging procedure				
Sentinel-node biopsy	15 (31.3)	18 (40.9)	0.931	0.335
Axillary dissection	33 (68.7)	26 (59.1)	0.931	0.335
Pathological staging				
I	20 (41.7)	16 (36.4)	0.271	0.603
II	23 (47.9)	21 (47.7)	0.000	0.986
III	5 (10.4)	7 (15.9)	0.611	0.435

Molecular subtype				
Luminal A	9 (18.8)	10 (22.7)	0.222	0.638
Luminal B (HER2 negative)	15 (31.2)	9 (20.5)	1.388	0.239
Luminal B (HER2 positive)	6 (12.5)	7 (15.9)	0.220	0.639
HER2 positive	12 (25.0)	9 (20.5)	0.269	0.604
Basal-like	6 (12.5)	9 (20.5)	1.604	0.302
Blood test				
WBC (x10^9/L)	6.4±1.5	6.2±1.3	0.822	0.413
ANC (x10^9/L)	4.3±1.4	3.9±1.0	1.642	0.104
HBG (g/L)	122.8±20.7	124.1±12.1	-0.353	0.725
PLT (x10^9/L)	213.8±53.1	201.0±43.2	1.263	0.210
Liver and kidney functions				
ALT (U/L)	21.6±25.7	17.7±9.1	0.952	0.344
AST (U/L)	19.5±4.6	19.2±5.6	0.217	0.829
GGT (U/L)	25.8±17.2	24.6±19.7	0.307	0.760
sCr (μmol/L)	52.1±13.6	51.4±10.8	0.261	0.795
Blood lipids				
TG (mmol/L)	1.2±0.7	1.6±1.0	-1.966	0.053
TC (mmol/L)	4.9±1.1	4.7±1.2	0.726	0.470
Cardiac function				
CK (U/L)	77.0±35.5	76.2±29.4	0.107	0.915
LVEF (%)	71.0±6.2	69.6±5.4	0.866	0.391

Variables are expressed as either mean±standard deviation or number of case (percentage).

Supplementary Table 3: Comparison of myelosuppression occurred during chemotherapy between the two groups

Group	White blood cell reduction grading					*Z*	*P*
	Grade 0	Grade 1	Grade 2	Grade 3	Grade 4		

MOX	3	7	21	17	0	-1.196	0.232
CON	1	6	16	21	0		
	Neutrophil reduction grading						
	Grade 0	Grade 1	Grade 2	Grade 3	Grade 4		
MOX	0	0	18	19	11	-0.662	0.508
CON	2	3	6	19	14		
	Hemoglobin reduction grading						
	Grade 0	Grade 1	Grade 2	Grade 3	Grade 4		
MOX	12	19	15	2	0	-1.073	0.283
CON	6	19	18	1	0		
	Platelet reduction grading						
	Grade 0	Grade 1	Grade 2	Grade 3	Grade 4		
MOX	45	3	0	0	0	-1.229	0.219
CON	38	4	2	0	0		

Supplementary Table 4: Comparison of the WBC count reduction grading between the two groups

Chemotherapy course	Group	n	WBC count reduction grading			
			Grade 0	Grade 1	Grade 2	Grade 3
1st course	MOX	48	13 (27.1)	11 (22.9)	16 (33.3)	8 (16.7)
	CON	44	11 (25.0)	6 (13.6)	17 (38.6)	10 (22.7)
2nd course	MOX	48	11 (22.9)	20 (41.7)	13 (27.1)	4 (8.3)
	CON	44	10 (22.7)	13 (29.5)	17 (38.6)	4 (9.1)
3rd course	MOX	48	13 (27.1)	13 (27.1)	15 (31.3)	7 (14.6)
	CON	43	12 (27.9)	12 (27.9)	13 (30.2)	6 (14.0)
4th course	MOX	42	16 (38.1)	8 (19.0)	13 (31.0)	5 (11.9)
	CON	37	13 (35.1)	9 (24.3)	9 (24.3)	6 (16.2)
5th course	MOX	34	22 (64.7)	9 (26.5)	2 (2.9)	1 (2.9)

	CON	29	18 (54.5)	7 (21.2)	2 (6.1)	2 (6.1)
6th course	MOX	40	23 (57.5)	6 (15.0)	8 (20.0)	3 (7.5)
	CON	38	23 (60.5)	7 (18.4)	4 (10.5)	4 (10.5)
7th course	MOX	22	12 (54.5)	8 (36.4)	1 (4.5)	1 (4.5)
	CON	24	11 (45.8)	4 (16.7)	5 (20.8)	4 (16.7)
8th course	MOX	13	7 (53.8)	5 (38.5)	1 (7.7)	0 (0.0)
	CON	12	4 (33.3)	5 (41.7)	1 (8.3)	2 (16.7)

Variables are expressed as number of case (percentage).

Supplementary Table 5: Comparison of the ANCs at specific time points between the two groups

Chemotherapy course	Group	n	ANC		
			1st week after chemotherapy (1-7d)	2nd week after chemotherapy (8-14d)	3rd week after chemotherapy (15-20d)
1st course	MOX	48	3.12±1.23 (37)	1.31±1.24 (44)	4.31±1.62 (47)
	CON	44	3.45±1.54 (35)	1.18±0.95 (40)	4.013±2.14 (44)
2nd course	MOX	48	3.55±1.28 (39)	1.57±1.00 (45)	3.26±1.50 (46)
	CON	44	3.89±2.02 (32)	1.40±0.75 (37)	3.71±1.63 (44)
3rd course	MOX	48	3.19±1.38 (37)	1.60±1.08 (46)	3.17±1.45 (47)
	CON	44	3.25±1.64 (32)	1.49±1.05 (38)	3.42±1.36 (42)
4th course	MOX	48	3.52±1.76 (27)	1.85±1.32 (36)	3.25±1.22 (39)
	CON	44	3.68±1.51 (21)	1.75±1.77 (30)	3.76±1.51 (35)
5th course	MOX	33	3.26±1.77 (23)	3.02±1.33 (29)	4.34±1.53 (33)
	CON	34	3.87±2.20 (19)	2.66±1.77 (25)	3.92±1.47 (33)
6th course	MOX	33	3.44±1.42 (18)	3.00±1.42 (24)	3.74±1.45 (24)
	CON	34	2.84±1.90 (16)	2.41±1.50 (19)	3.60±1.28 (24)
7th course	MOX	20	3.31±1.48 (14)	2.90±1.73 (19)	3.20±0.93 (20)
	CON	23	2.57±1.54 (13)	2.58±1.41 (19)	3.47±1.10 (21)

8th course	MOX	20	2.93±1.14 (9)	2.77±1.00 (9)	3.70±1.23 (8)
	CON	23	2.95±1.76 (6)	2.92±1.98 (7)	3.06±1.26 (6)

ANCs are expressed as mean±standard deviation x10^9/L (number of case).

Supplementary Table 6: Comparison of adverse effects between the two groups

	MOX (n=48)	CON (n=44)
Liver and kidney functions		
ALT elevation	20 (41.7)	13 (29.5)
Grade 1	18 (37.5)	11 (25.0)
Grades 2-3	2 (4.2)	2 (4.5)
AST elevation	18 (37.5)	14 (31.8)
Grade 1	16 (33.3)	11 (25.0)
Grades 2-3	2 (4.2)	3 (6.8)
GGT elevation	24 (50.0)	23 (52.3)
Grade 1	15 (31.3)	18 (40.9)
Grade 2	9 (18.8)	5 (11.4)
sCr elevation	0 (0.0)	0 (0.0)
Blood lipids		
TG elevation	27 (56.3)	30 (68.2)
Grade 1	21 (43.8)	19 (43.2)
Grade 2	6 (12.5)	11 (25.0)
TC elevation	20 (41.7)	16 (36.4)
Grade 1	19 (39.6)	14 (31.8)
Grade 2	1 (2.1)	2 (4.5)
Cardiac toxicity		
CK elevation	0 (0.0)	0 (0.0)
LVEF reduction	9 (18.8)	9 (20.5)
Grade 1	2 (4.2)	5 (11.4)

Grade 2	7 (14.6)	4 (9.1)

Variables are expressed as number of case (percentage).

Supplementary Table 7: Comparison of systemic general adverse effects between the two groups

Systemic general adverse effects	MOX (n=48)	CON (n=44)	Difference in percentage	χ^2	*P*
Fatigue					
Grade 1	42 (87.5)	41 (93.2)	-5.7	0.840	0.359
Grade 2	6 (12.5)	9 (20.5)	-8.0	1.064	0.302
Malaise					
Grade 1	41 (85.4)	40 (90.9)	-5.5	0.658	0.417
Grade 2	4 (8.3)	7 (15.9)	-7.6	1.252	0.263
Weight loss	6 (12.5)	5 (11.4)	+1.1	0.028	0.867

Variables are expressed as number of case (percentage).

Supplementary Table 8: Comparison of gastrointestinal adverse effects between the two groups

Gastrointestinal reactions	MOX (n=48)	CON (n=44)	Difference in percentage	χ^2	*P*
Nausea	39 (81.3)	36 (81.8)	-0.5	0.005	0.944
Grade 1	26 (54.2)	15 (34.1)	+20.1	3.745	0.053
Grades 2-3	13 (27.1)	21 (47.7)	-20.6	4.199	0.040*
Vomiting	27 (56.3)	30 (68.2)	-11.9	1.387	0.239
Grade 1	11 (22.9)	8 (18.2)	+4.7	0.314	0.575
Grades 2-3	16 (33.3)	22 (50.0)	-16.7	2.630	0.105
Diarrhea	10 (20.8)	15 (34.1)	-13.3	2.039	0.153
Grade 1	7 (14.6)	10 (22.7)	+8.1	1.011	0.315
Grade 2	3 (6.3)	5 (11.4)	-5.1	0.756	0.385
Constipation	23 (47.9)	24 (54.5)	-6.6	0.404	0.525
Grade 1	22 (45.8)	23 (52.3)	-6.5	0.381	0.537
Grade 2	1 (2.1)	1 (2.3)	-0.2	0.004	1.000

Variables are expressed as number of case (percentage).

*P<0.05.

Supplementary Table 9: Comparison of the pain adverse effects between the two groups

Pain	MOX (n=48)	CON (n=44)	Difference in percentage	χ^2	*P*
General pain	43 (89.6)	43 (97.7)	-8.1	2.497	0.206
Musculoskeletal and connective tissue disorders	21 (43.8)	29 (65.9)	-22.1	4.543	0.033*
Incision pain	5 (10.4)	12 (27.3)	-16.9	4.330	0.037*
Headache	8 (16.7)	11 (25.0)	-8.3	0.973	0.324
Gastrointestinal pain	5 (10.4)	4 (9.1)	+1.3	0.046	1.000
Breast pain	4 (8.3)	3 (6.8)	+1.5	0.075	1.000
Toothache	1 (2.1)	3 (6.8)	-4.7	1.238	0.346
Pharyngolaryngeal pain (Throat pain)	3 (6.3)	1 (2.3)	+4.0	0.873	0.618

Variables are expressed as number of case (percentage).

Musculoskeletal and connective tissue disorders include bone pain, arthralgia, and muscle pain.

**P*<0.05.

Supplementary Table 10: Comparison of the other adverse effects between the two groups

Other adverse effects	MOX (n=48)	CON (n=44)	Difference in percentage	χ^2	*P*
Hot flashes	12 (25.0)	5 (11.4)	+13.6	2.834	0.092#
Dizziness	2 (4.2)	12 (27.3)	-23.1	9.500	0.002**
Dry mouth	18 (37.5)	16 (36.4)	+1.1	0.013	0.910
Insomnia	17 (35.4)	16 (36.4)	-1.0	0.009	0.925
Infections[1]	9 (18.8)	13 (29.5)	-10.7	1.470	0.225
Hyperhidrosis (sweating)	12 (25.0)	8 (18.2)	+6.8	0.627	0.428
Pruritus	3 (6.3)	5 (11.4)	-5.1	0.756	0.473
Palpitations	5 (10.4)	3 (6.8)	+3.6	0.374	0.716
Paresthesia[2]	4 (8.3)	2 (4.5)	+3.8	0.540	0.679
Abdominal distension	3 (6.3)	3 (6.8)	-0.5	0.012	1.000
Edema[3]	2 (2.6)	3 (6.8)	-4.2	0.314	0.667

Hemorrhage[4]	2 (4.2)	1 (2.3)	+1.8	0.261	1.000
Nail discoloration	2 (4.2)	1 (2.3)	+1.9	0.261	1.000
Tinnitus	1 (2.1)	2 (4.5)	-2.4	0.441	0.605
Surgical and medical procedures[5]	3 (6.3)	0 (0.0)	+6.3	2.843	0.243

Variables are expressed as number of case (percentage).

#P=0.092[#], **P<0.01.

[1]Infection includes oral mucositis, rhinitis, pneumonia, cholecystitis, gastroenteritis, pelvic inflammatory disease, incision infection, and urinary tract infection.

[2]Paresthesia includes dysesthesia, dysgeusia, scalp numbness, and general numbness.

[3]Edema includes facial edema, pedal edema, and periocularedema.

[4]Hemorrhage includes bleeding gums, nosebleed, and subconjunctival hemorrhage.

[5]Surgical and medical procedures include 1 case of venous thrombosis and 2 cases of breast surgery.

Supplementary Table 11: Comparison of the chemotherapy compliance between the two groups

	MOX (n=48)	CON (n=44)	χ^2	P
FN	1 (2.1)	6 (13.6)	4.359	0.051
Grades 3-4 myelosuppression	30 (62.5)	36 (81.8)	4.226	0.040
Grade 3biochemical indicator	10 (20.8)	12 (27.3)	0.523	0.469
Delayed chemotherapy	9 (18.8)	11 (25.0)	0.527	0.468
Reduced chemotherapy	1 (2.1)	5 (11.4)	3.243	0.100

Variables are expressed as number of case (percentage).

Supplementary Table 12: Clinical characteristics of the 6 cases with survival events observed

Patient ID	A	B	C	D	E	F
Number	29	32	49	65	89	92
Group	MOX	MOX	CON	CON	CON	MOX
Age	57	61	33	53	56	26
Menstrual status	Menopause	Menopause	Not menopause	Not menopause	Menopause	Not menopause
Marital status	Married	Married	Married	Married	Married	Unmarried
Clinical staging	IIIA	I	IIIC	IIA	I	IIB
Molecular typing	HER2positive	Basal-like	Luminal B (HER2 negative)	Luminal B(HER2 negative)	Luminal B (HER2positive)	Luminal B (HER2negative)
Breast-conserving	Yes	No	No	No	No	No
Axilla-conserving	No	Yes	No	Yes	Yes	No
Chemotherapy regimen	Anthracycline-taxne	Anthracycline-taxne	Anthracycline-taxne	Anthracycline-taxne	Anthracycline-taxne	Anthracycline-taxne
Specific medication and treatment	EC×4-T×4	EC×4-T×4	EC×4-T×4	CEF×3-T×3	EC×4-TH×4	CEF×3-T×3
Radiotherapy	Yes	No	Yes	No	No	Yes
Endocrine therapy	None	AI	OFS+AI	TAM	AI	TAM
TCM treatment during rehabilitation	No	Yes	Yes	Yes	Yes	No
Inadequate treatment	Reject H due to economic reasons	None	None	None	None	Reject OFS due to economic reasons
DFS event	Lung metastasis	Chest wall recurrence<br>Lung metastasis	Lung, liver and bone metastasis	Liver metastasis	Lung metastasis	Lung metastasis
DFS (months)	13.0	21.8	21.5	25.2	24.0	25.1
OS event	None	Respiratory failure	Respiratory failure	None	None	None
OS (months)	47.7	31.1	33.2	43.1	39.2	39.1

Death interval (months)*	-	9.3	11.7	-	-	-

*Death interval is defined as the time between distant recurrence and death.

Abbreviation: E, epirubicin; C, cyclophosphamide; T, taxol; F, fluorouracil; H, herceptin; AI, aromatase inhibitor; OFS, ovarian function suppression; TAM, tamoxifen.
